# Congenital Vitelline Band Causing Intestinal Obstruction in an Adult with a Double Inferior Vena Cava

**DOI:** 10.1155/2016/4015408

**Published:** 2016-10-23

**Authors:** Mihiri Wettasinghe, Kumari Pussepitiya, Bandula Samarasinghe, Nuwan Wickramasinghe

**Affiliations:** ^1^Department of Radiology, Teaching Hospital, Peradeniya, Sri Lanka; ^2^Department of Surgery, Faculty of Medicine, University of Peradeniya, Peradeniya, Sri Lanka; ^3^Department of Community Medicine, Faculty of Medicine and Allied Sciences, Rajarata University of Sri Lanka, Saliyapura, Sri Lanka

## Abstract

*Introduction*. Vitelline artery remnants are rare causes of intra-abdominal bands leading to bowel obstruction. These bands may be associated with Meckel's diverticulum. Double inferior vena cava (IVC) is a rare presentation and is usually identified incidentally.* Case Presentation*. A sixty-year-old male presented with progressive vomiting for five days and he was clinically diagnosed with intestinal obstruction. Plain X-ray abdomen showed evidence of small bowel obstruction. CT scan of the abdomen revealed dilated small bowel loops with a small outpouching in the distal ileum with a band like structure attached to it. In the CT, left sided patent IVC draining into the left renal vein was identified. Left external iliac vein was in continuity with the left IVC. Left internal iliac vein was draining into the right IVC. Exploratory laparotomy revealed a Meckel's diverticulum with a band identified as the vitelline remnant attached to its apex and inserting at the anterior abdominal wall near the umbilicus.* Discussion*. Meckel's diverticulum with vitelline bands, although rare, should be borne in mind in adult patients with intestinal obstruction. Identification of this anomaly can be difficult in imaging studies. Presence of double IVC should be mentioned in the imaging findings to prevent possible catastrophic complications during surgery.

## 1. Introduction

Meckel's diverticulum is the commonest congenital anomaly in the gastrointestinal system, though being most often detected as an incidental finding. Incomplete obliteration of the omphalomesenteric duct is considered as the cause for this anomaly. However, apart from Meckel's diverticulum, there is a spectrum of anomalies that arise as a result of complete or incomplete obliteration of the omphalomesenteric duct. These include umbilicoileal fistula, omphalomesenteric duct sinus, omphalomesenteric duct cyst, and fibrous connection of the ileum to the umbilicus [1]. As the vitellointestinal duct connects the developing midgut to the yolk sac in the embryo, incomplete resorption of the obliterated vitelline duct results in fibrous band connecting the distal ileum to the umbilicus. Although, in the majority, Meckel's diverticulum does not lead to any complications, approximately 4% of the affected individuals develop complications related to Meckel's diverticulum including haemorrhage, perforation, diverticulitis, and obstruction [2]. Double IVC is also a rare presentation and is usually identified incidentally. The present case illustrates an old male presenting with intestinal obstruction due to vitelline band with a Meckel's diverticulum and incidental finding old double IVC in the same patient.

## 2. Case Presentation

A 69-year-old previously healthy male patient presented with a history of vomiting for five days' duration. The vomitus contained clear fluid initially, which later became brownish in colour. He had developed absolute constipation for three days. He had noticed gradual abdominal distension during this period. He had not undergone any abdominal surgeries in the past.

On examination, his abdomen was distended and there was mild tenderness in the lower abdomen. The examination of the respiratory system did not reveal any abnormalities. Biochemical investigations were unremarkable. With the clinical diagnosis of intestinal obstruction, he was referred for imaging studies.

### 2.1. X-Ray Abdomen

**Figure 1:**
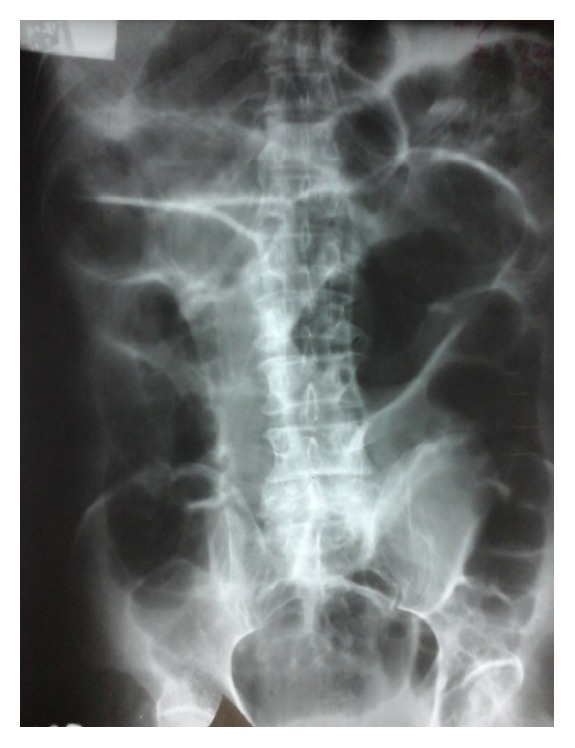
Supine X-ray abdomen showing dilated small bowel loops.

Plain X-ray abdomen was performed in the supine position and it revealed grossly distended small bowel loops with absent rectal gas (Figure 1). There was no pneumoperitoneum.

### 2.2. USS Abdomen

Ultrasound scan of the abdomen revealed fluid filled aperistaltic bowel loops. Central abdomen was distended and presence of bowel gas hindered evaluating the deeper structures. Pancreas appeared normal. There was no free fluid in the abdomen. Liver, spleen, and both kidneys were normal on ultrasound. The superior mesenteric artery appeared normal at the origin and showed normal colour and spectral flow pattern. The portal vein was normal.

### 2.3. CT Abdomen

**Figure 2:**
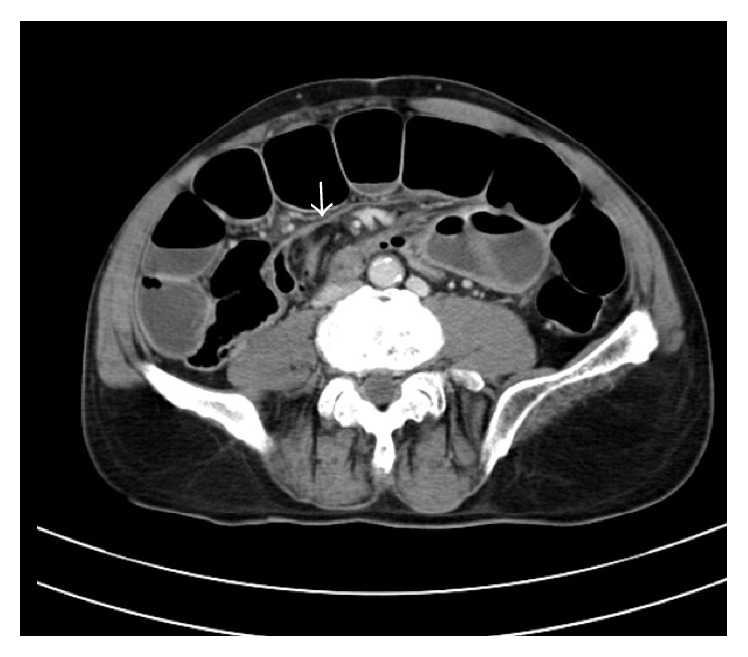
CT abdomen axial view showing band like structure extending from the distal ileum (white arrow).

CT abdomen showed grossly dilated jejunal and ileal loops. The appendix was identified separately. There were no fat strandings or fluid collections around the appendix. Distal ileal loops appeared collapsed. There was a soft tissue density band like structure extending from the collapsed ileal loops anteriorly (Figure 2).

However, its insertion site was not identified. There was no CT evidence of appendicitis. The cause for the small bowel obstruction was not identified on CT. However, as the patient had no previous surgeries, the possibility of a congenital band was highly suspected. The presence of a soft tissue density linear band led to the preoperative suspicion of a congenital band. Furthermore, Meckel's diverticulum was not considered on CT images.

**Figure 3:**
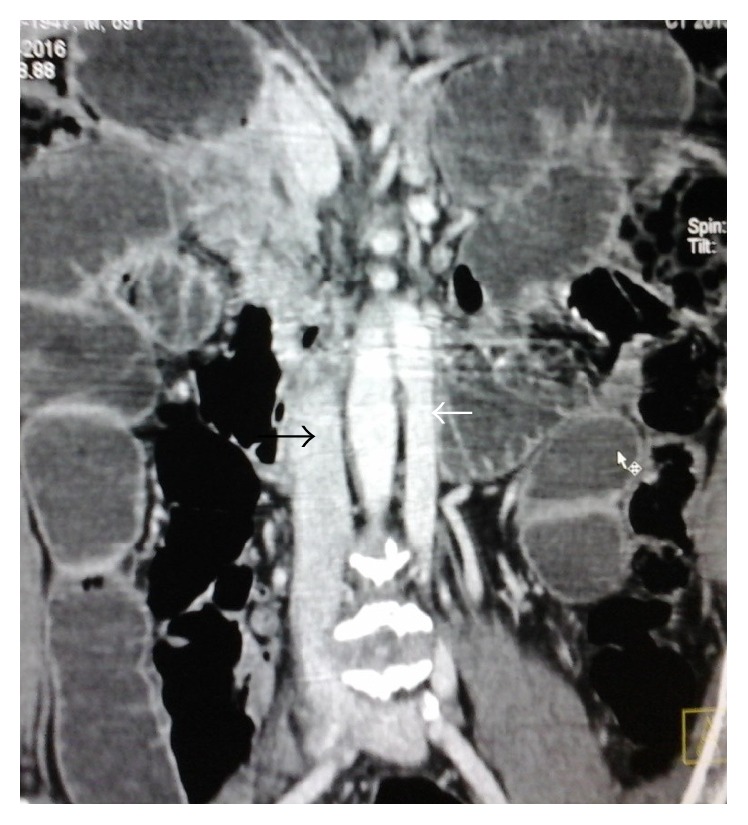
Coronal CT image showing double IVC with small left IVC (white arrow) and large caliber right IVC (black arrow).

There was a tubular retroperitoneal structure in the left side of the aorta which was extending up to the left renal vein. It was in continuity with the left external iliac vein and was identified as the left IVC. Right IVC was also noted. Left IVC was smaller in caliber than the right IVC (Figure 3).

**Figure 4:**
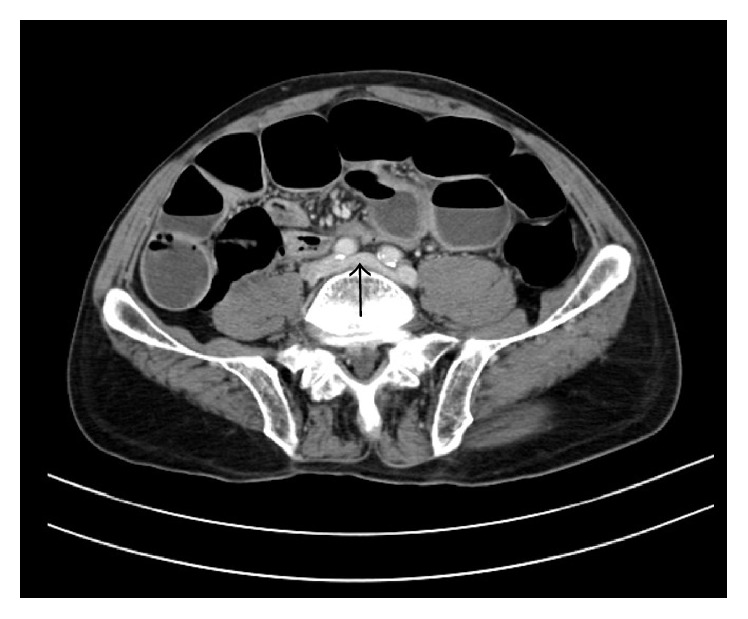
Left internal iliac vein draining to right common iliac vein (black arrow).

Left internal iliac vein was seen crossing the midline to drain into the right common iliac vein (Figure 4).

## 3. Surgical Findings

**Figure 5:**
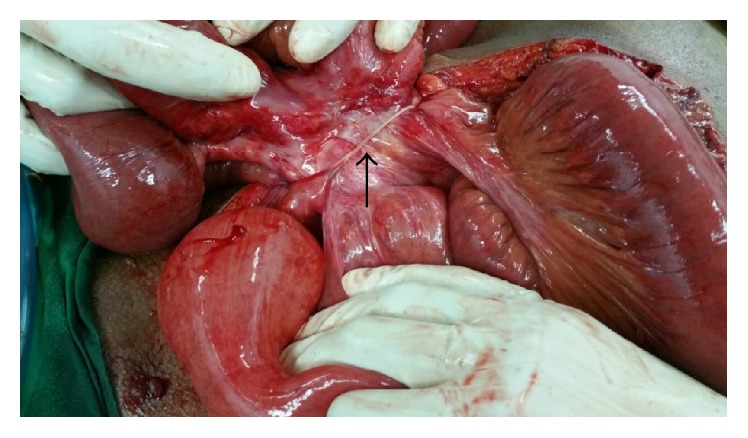
Fibrous band extending from Meckel's diverticulum to anterior abdominal wall (black arrow).

Patient underwent exploratory laparotomy. There was a Meckel's diverticulum with fibrous band extending to the anterior abdominal wall (Figure 5). The size of the diverticulum was approximately 3 cm. The band was identified as the obliterated vitelline duct. The diverticulum was seen about 20 cm proximal to the ileocecal valve, arising from the antimesenteric border of the distal ileum. The small bowel loops were twisting around the band forming a volvulus, causing the small bowel obstruction. The band was resected and the obstruction was relieved.

## 4. Discussion 

Meckel's diverticulum is the result of incomplete obliteration of the vitelline or omphalomesenteric duct and is the commonest congenital anomaly of the gastrointestinal system [3]. It is located in the antimesenteric border of the distal ileum and is considered to be a true diverticulum [4]. As stated in the literature, intestinal obstruction secondary to Meckel's diverticulum is difficult to identify preoperatively [1] and this was seen in our patient as well.

Most of the instances, this anomaly is asymptomatic and the lifetime risk of developing complications is between 4% and 6% [5]. Furthermore, the presence of complications in elderly patients is even rare. Thus, developing small bowel obstruction from Meckel's diverticulum in an elderly person, as seen in our patient, is relatively rare. Haemorrhage, obstruction, and inflammation are considered to be the most frequent complications of Meckel's diverticulum and obstruction can be due to trapping of a bowel loop by a mesodiverticular band, a volvulus of the diverticulum around a mesodiverticular band, and intussusception [3, 4]. In our patient, the cause of obstruction was the vitelline band. Although a soft tissue density linear band was seen extending from the distal ileum, its insertion at the anterior abdominal wall was not identified. This could have been the reason for not diagnosing the vitelline band in the CT images.

Small intestine obstruction due to persistent vitelline-intestinal duct is extremely rare, especially in adult patients, and very few cases were reported in the literature [6]. Thus, age of the patient precluded preoperative diagnosis of Meckel's diverticulum and vitelline band. As these were not evident on CT images, definitive diagnosis for the cause of the obstruction was determined during the surgery. Although the cause for the obstruction is not clearly depicted in the CT images, early surgical intervention is vital in the management of these patients, as the cause for the obstruction is mechanical and delay in surgery would result in bowel ischaemia and gangrene.

Anatomical variation of the inferior vena cava occurs in 0.4–4% of the population [7]. Formation of IVC is a complex event that occurs in early embryonic life and adult form of IVC is the result of formation of series of anastomoses and regression of venous structures. Persistence of left supracardinal vein along with the right one leads to double IVC anomaly [7, 8]. Left IVC drains into left renal vein, which in turn drains into right IVC, forming a single suprarenal IVC. The left IVC may drain both the internal and external iliac veins in the left side. Sometimes, only the external iliac vein drains into left IVC and the internal iliac vein crosses the midline to drain into the right common iliac vein [9]. This variant was seen in our patient.

The importance of identifying the left IVC is that it can be mistaken for lymph node mass. It is also important to inform the referring surgeon regarding this anomaly to avoid catastrophic complications during surgery [10]. Although double IVC is known to be associated with a range of genitourinary abnormalities including horseshoe kidney, crossed fused ectopia, cloacal exstrophy, and retroaortic renal vein, none of these anomalies were seen in our patient [11]. There are reports on association between double IVC and congenital heart disease [12].

Reports on association of double IVC and congenital gastrointestinal anomalies are scarce. One case report was found in the literature stating duplication of inferior vena cava and malrotation of gut in the same patient [13]. However, meticulous literature search did not reveal any evidence on association between double IVC and congenital vitelline band.

## 5. Conclusion

In conclusion, although not common, Meckel's diverticulum should be borne in mind when elderly patients present with intestinal obstruction. Identification of this anomaly can be difficult in imaging studies. Presence of double IVC should be mentioned in the imaging findings as this will prevent catastrophic complications during surgery. It is important to communicate the presence of a double IVC to the operating surgeon, in order to avoid forceful interference during the surgical procedure.
